# Transcriptome-wide association study reveals cholesterol metabolism gene Lpl is a key regulator of cognitive dysfunction

**DOI:** 10.3389/fnmol.2022.1044022

**Published:** 2022-12-15

**Authors:** Wei Hu, Jian Liu, Yaorui Hu, Qingling Xu, Tingzhi Deng, Mengna Wei, Lu Lu, Jia Mi, Jonas Bergquist, Fuyi Xu, Geng Tian

**Affiliations:** ^1^School of Chemical Engineering and Technology, Tianjin University, Tianjin, China; ^2^Shandong Technology Innovation Center of Molecular Targeting and Intelligent Diagnosis and Treatment, School of Pharmacy, Binzhou Medical University, Yantai, Shandong, China; ^3^Department of Plastic Surgery, The First Affiliated Hospital of Shandong First Medical University, Shandong Provincial Qianfoshan Hospital, Jinan, Shandong, China; ^4^Jinan Clinical Research Center for Tissue Engineering Skin Regeneration and Wound Repair, Jinan, Shandong, China; ^5^Department of Ultrasound, Yantai Affiliated Hospital, Binzhou Medical University, Yantai, Shandong, China; ^6^Department of Genetics, Genomics, and Informatics, The University of Tennessee Health Science Center, Memphis, TN, United States; ^7^Department of Chemistry - BMC, Uppsala University, Uppsala, Sweden

**Keywords:** BXD mice, cognitive dysfunction, Lpl, genetic regulation, hippocampus

## Abstract

Cholesterol metabolism in the brain plays a crucial role in normal physiological function, and its aberrations are associated with cognitive dysfunction. The present study aimed to determine which cholesterol-related genes play a vital role in cognitive dysfunction and to dissect its underlying molecular mechanisms using a systems genetics approach in the BXD mice family. We first systematically analyzed the association of expression of 280 hippocampal genes related to cholesterol metabolism with cognition-related traits and identified lipoprotein lipase (Lpl) as a critical regulator. This was further confirmed by phenome-wide association studies that indicate Lpl associated with hippocampus volume residuals and anxiety-related traits. By performing expression quantitative trait locus mapping, we demonstrate that *Lpl* is strongly *cis-*regulated in the BXD hippocampus. We also identified ∼3,300 genes significantly (*p* < 0.05) correlated with the *Lpl* expression. Those genes are mainly involved in the regulation of neuron-related traits through the MAPK signaling pathway, axon guidance, synaptic vesicle cycle, and NF-kappa B signaling pathway. Furthermore, a protein–protein interaction network analysis identified several direct interactors of Lpl, including Rab3a, Akt1, Igf1, Crp, and Lrp1, which indicates that Lpl involves in the regulation of cognitive dysfunction through Rab3a-mediated synaptic vesicle cycle and Akt1/Igf1/Crp/Lrp1-mediated MAPK signaling pathway. Our findings demonstrate the importance of the Lpl, among the cholesterol-related genes, in regulating cognitive dysfunction and highlighting the potential signaling pathways, which may serve as novel therapeutic targets for the treatment of cognitive dysfunction.

## Highlights

- This is the first study to systemically assess the associations between cholesterol metabolism-related genes and cognitive functions.
- Among the 280 cholesterol metabolism-related genes, we found that decreased *Lpl* expression is associated with impaired cognitive function, as well as emotional and anxious behaviors, through modulation of synaptic vesicles and MAPK signaling pathways.
- This finding demonstrates that Lpl is a crucial regulator of cognitive function and may serve as a novel therapeutic target for the treatment of cognitive dysfunction.

## Introduction

The cholesterol metabolism maintains crucial physiological functions in the brain (Zhang and Liu, 2015). Dysfunction of this pathway is associated with various neurological and neurodegenerative disorders, including Alzheimer’s, Huntington’s, and Parkinson’s diseases (Pfrieger, 2021). Even though the detailed mechanism remains largely unknown, several recent studies have confirmed the causal relationship between cholesterol metabolism and cognitive function (Djelti et al., 2015). However, cholesterol metabolism is a complicated process including over 200 genes involved in cholesterol synthesis, transportation, uptake, storage, and release (Martín et al., 2014), how these genes are systematically associated with cognitive function is still unknown.

With the development of high throughput sequencing technology, transcriptome-wide association study (TWAS) has become a powerful approach to establishing the association between gene expression and phenotypes at a population level (Wainberg et al., 2019). Establishing a gene–gene and gene–phenotype correlation network makes it possible to identify potential key regulators and mechanisms. Moreover, with the further combination of expression quantitative trait locus (eQTL) analysis, it is even possible to reveal the potential genetic regulation of certain genes. Nevertheless, such analysis in neurological studies requires assessing the gene expression of central nervous system (CNS) tissue, which is not practical in the human population (Li and Auwerx, 2020). The mouse genetic reference population has been extensively used for genetics studies in neurodegenerative disorders (Li, 2019). The BXD mice family contains over 150 recombinant inbred strains, which descended from the hybridization between inbred strains C57BL/6J (B6) and DBA/2J (D2) with stable genetic variations (Ashbrook et al., 2021). In addition, dozens of transcriptomes across various CNS tissues have been generated. Combing these data with the cognition phenotypes makes it a unique resource to perform a TWAS study in neurological study.

This study aimed to explore the cholesterol-related gene expression associated with cognitive dysfunction and its underlying mechanism with a TWAS study based on the BXD population. The result reveals the Lpl expression level in the hippocampus is significantly correlated with the learning and memory function. Moreover, the *Lpl* expression is strongly genetic *cis*-regulated, and the potential of the mechanism is discussed.

## Materials and methods

### Hippocampus transcriptomic data set

The hippocampus is an important and intriguing part of the forebrain that is crucial in memory formation and retrieval and is often affected in epilepsy, Alzheimer’s disease, and schizophrenia. The BXD hippocampus transcriptomic data set used in this study provides estimates of mRNA expression in the adult hippocampus of 67 BXD recombinant inbred strains, two parental strains (C57BL/6J and DBA2/J), and two reciprocal F1 hybrids. The raw microarray data are available on GEO^1^ under the identifier GSE84767. The normalized data set “hippocampus Consortium” is available on the GeneNetwrok (Mulligan et al., 2017) under the “BXD” group and “hippocampus mRNA” type with the identifier “Hippocampus Consortium M430v2 (June 06) RMA”. Below are brief descriptions of how this data set was generated.

#### Mice and tissue harvesting

Animals from 71 BXD strains were obtained and housed at the UTHSC under the controlled breeding environment with 40∼60% humanity and 18∼22°C. Mice were euthanized by cervical dislocation at the age of 45–90 days. Brains were removed and placed in RNA later, and the whole hippocampi were dissected. All procedures involving mouse tissue were approved by the Institutional Animal Care and Use Committee at the University of Tennessee Health Science Center.

#### RNA extraction and evaluation

A pool of dissected tissue, typically from six hippocampi and three naive adults of the same strain, sex, and age, was collected in one session and used to generate cRNA samples. A total of 143 RNA samples were extracted with RNA STAT-60 according to the manufacturer’s instructions. This includes tissue homogenization, RNA extraction, precipitation, and wash. The RNA was further purified with Na4OAc, and its purity and integrity were evaluated using the 260/280 nm absorbance ratio and the Agilent Bioanalyzer 2100, respectively. RNA with 260/280 values greater than 1.8 and RNA integrity numbers greater than 8 are required to run the array.

#### Microarray and data normalization

Pooled RNA samples from two to three animals were hybridized into a single Affymetrix GeneChip Mouse Expression 430 2.0 short oligomer arrays. Raw microarray data were normalized using the RMA methods (Bolstad et al., 2003) and further transformed with a modified z-score (2z + 8) (Chesler et al., 2005). This analysis was done with R statistical functions. The detailed sample info is listed in Supplementary Table 1.

### eQTL mapping

For the analyses presented here, whole genome eQTL mapping was carried out using 71 BXD strains on GeneNetwork with a modified Haley–Knott regression (Haley and Knott, 1992). The resulting likelihood ratio statistic (LRS) was used to evaluate the associations between the genotypes^2^ and gene expression levels. Genome-wide significant and suggestive thresholds were determined with 1,000 permutation tests. This analysis was done on GeneNetwork.

### Sequence variants

Genetic variations between parental strains B6 and D6 were searched with our previous whole genome resequencing data and Mouse Genome Project^3^ (Keane et al., 2011).

### Correlation analysis

The Pearson correlation coefficient analysis was deployed to identify the covariates of the gene of interest and the BXD-published traits/phenotypes. A *p*-value lower than 0.05 is considered statistically significant. This analysis was done on the GeneNetwork.

### Gene function enrichment analysis

A gene function enrichment analysis was done with a hypergeometric test on the WEB-based Gene SeT AnaLysis Toolkit (WebGestalt)^4^ (Liao et al., 2019). The resulting False Discovery Rate (FDR) lower than 0.05 was used to define the overrepresented terms, including the KEGG pathway and gene ontology (GO).

### Phenome-wide association analysis (PheWAS)

We used the SNP genotypes (missense, splice site, and *cis-*eQTL variants) within the Lpl gene to perform PheWAS against 5,000 clinical phenotypes in the BXD population (Li et al., 2018). The multi-locus mixed-model approach (mlmm) was used to estimate the associations between Lpl and clinical phenotypes. The kinship matrix from BXD strains was applied to account for the population structure. The Bonferroni’s method was used to correct the multiple testing. The clinical phenome is currently split into 13 broad categories based on general biological ontologies. This analysis was done on Systems Genetics and Omics Toolkit^5^ (Li et al., 2018).

## Results

### Correlation analysis reveals *Lpl* is a crucial regulator of cognitive function in BXD mice

**FIGURE 1:**
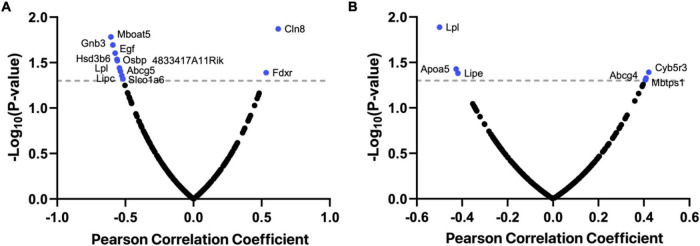
Bubble charts showing *Lpl* is a crucial regulator of cognitive function in BXD mice. The mRNA levels of 280 cholesterol-related genes in the BXD hippocampus were correlated against two cognition-related traits, Morris water maze performance **(A)** and y-maze performance **(B)**. Pearson correlation coefficient and -log10 *p*-value are indicated in the *x*- and *y*-axis. This analysis was done on the GeneNetwork (https://www.genenetwork.org/).

To determine which cholesterol-related genes play a pivotal role in cognitive function, we determined the expression levels of 280 genes linked to cholesterol metabolism across the BXD hippocampus. We performed correlation analysis against two cognition-related traits, Morris water maze performance and y-maze performance (Supplementary Table 2 and Figure 1). This gene list was compiled from the gene ontology database AmiGo 2^6^ by searching “cholesterol.” Among the 280 genes, 6 genes (*Lpl*, *Apoa5*, *Lipe*, *Cyb5r3*, *Abcg4*, and *Mbtps1*) were associated with Morris water maze performance (BXD_15171, *p* < 0.05), 12 genes (*Lpcat3*, *Gnb3*, *Hsd3b6*, *Egf*, *Abca12*, *Osbp*, *Lpl*, *Abcg5*, *Lipc*, *Slco1a6*, *Cln8*, and *Fdxr*) were associated with y-maze performance (BXD_20728, *p* < 0.05), and only one gene *Lpl* was significantly correlated with both investigated traits (*p* < 0.05, Figure 1).

### Lpl is associated with multiple cognition-related traits from both genotype and intermediate phenotype

**FIGURE 2:**
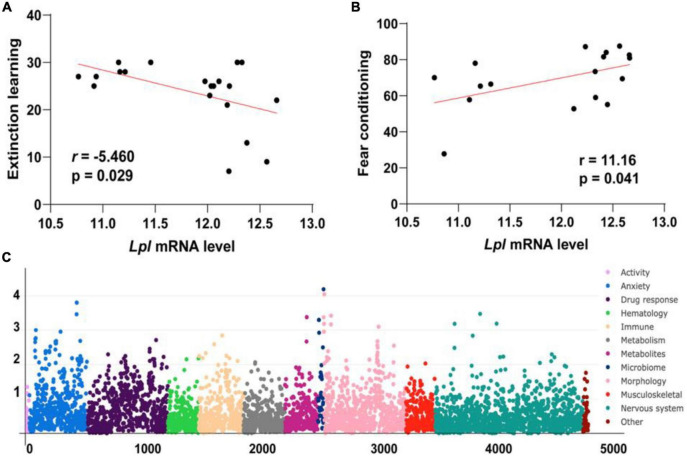
Lpl is associated with multiple cognition-related traits. Scatter plots of the correlations between the expression of Lpl and extinction learning **(A)** and fear conditioning **(B)**. The Pearson correlation coefficient was used to determine the relationship. Manhattan plots showing the Lpl-associated phenotypes in BXD mice **(C)**. This analysis was done on systems genetics (https://systems-genetics.org/) with the multi-locus mixed-model approach. The clinical phenome was grouped into 11 broad categories based on general biological ontologies, including activity, anxiety, drug response, hematology, immune, metabolism, metabolites, microbiome, morphology, musculoskeletal, and nervous system. *P*-values were adjusted with the Bonferroni’s method.

In addition to the associations with Morris water maze performance (*r* = −0.499, *p* = 0.013) and y-maze performance (*r* = −0.544, *p* = 0.036, Figure 1), *Lpl* mRNA levels were also found to be negatively correlated with extinction learning (*r* = −0.487, *p* = 0.029, Figure 2A) and positively related with fear conditioning (*r* = 0.498, *p* = 0.042, Figure 2B). Moreover, PheWAS between Lpl genotype and BXD phenome, comprising of ∼5,000 traits, revealed 19 traits showing moderate association (-log10(p) > = 3, Figure 2C and Table 1). This includes ventral hippocampus volume residuals and several anxiety assays, such as the time in open quadrants using an elevated zero maze.

**TABLE 1:** Lists of the 19 Lpl-associated phenotypes.

-Log10 (*p*-value)	Phenotype	Category
4.19538	Microbiome, Barnesiella (OTU) proportion assessed by 16S rRNA sequencing of fecal pellets from young adult males and females (residuals, log10 of fraction)	Microbiome
4.04995	Cerebellum internal granule layer (IGL) volume without adjustment (mm^3^)	Morphology
3.80718	Anxiety assay, restraint stress (15 min) and ethanol (1.8 g/kg ip) (RSE group), time in open quadrants using an elevated zero maze in 60–120-day-old males only during last 5 min (% time)	Anxiety
3.80435	Anxiety assay, restraint stress (15 min) and ethanol (1.8 g/kg ip) (RSE group), time in open quadrants using an elevated zero maze in 60–120-day-old males only during 10 min (% time)	Anxiety
3.47909	Neurexin 1 (Nrxn1) expression in hippocampus, first principal component generated using all coding exon probe sets and UMUTAffyExon_0209_RMA data (relative residual concentration)	Nervous system
3.46809	Anxiety assay, restraint stress (15 min) and ethanol (1.8 g/kg ip) (RSE group), time in open quadrants using an elevated zero maze in 60–120-day-old males only during first 5 min (% time)	Anxiety
3.42566	Hippocampus, ventral hippocampus volume residuals, adjusted for differences in age and brain weight (mm^3^)	Morphology
3.38372	Ratio of C18:2-carnitine/C18:1-carnitine_CD	Metabolites
3.37807	Cerebellum weight, whole, bilateral in adults of both sexes (mg)	Morphology
3.30292	Microbiome, Bacteroidales (order) frequency assessed by 16S rRNA sequencing of fecal pellets from young adult males and females (residuals, log10 of fraction)	Microbiome
3.30292	Microbiome, Bacteroidetes (class) frequency assessed by 16S rRNA sequencing of fecal pellets from young adult males and females (residuals, log10 of fraction)	Microbiome
3.19546	Basolateral amygdala residual volume, statistically adjusted for variation in sex, age, body weight (residual mm∧^3^)	Nervous system
3.18975	Amygdala, basolateral complex volume (LaDL, LaVL, LaVM, BLP, and BLA), unilateral shrinkage corrected and adjusted for variation in body weight and plane of section from serial histological sections (mm∧^3^)	Nervous system
3.18822	Cerebellum volume (mm^3^)	Morphology
3.17338	Hippocampus, ventral hippocampus volume, age-adjusted residuals (mm^3^)	Morphology
3.10528	Body weight gain between 9 and 10 weeks in males on high fat diet (45% energy from fat) feeding from 4 weeks on (g)	Morphology
3.00485	Novel open field behavior, urinations for males (n/test period)	Anxiety
2.95593	Brain weight, male and female adult average, unadjusted for body weight, age, sex (mg)	Morphology
2.95526	Anxiety assay, time in middle of an elevated plus maze for males and females (sec)	Anxiety

### The expression level of *Lpl* in the BXD hippocampus is strongly *cis*-regulated

**FIGURE 3:**
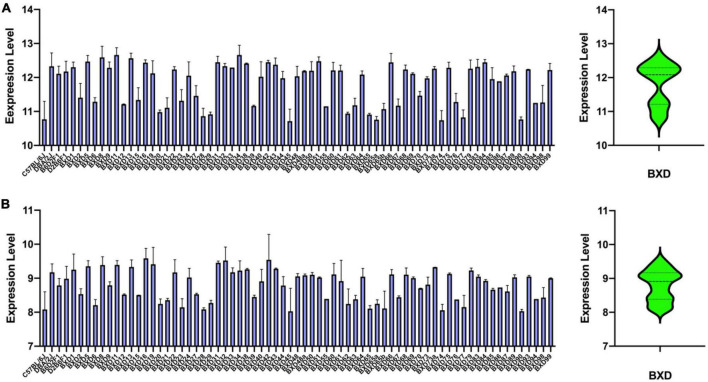
Bar and violin plots showing the *Lpl* expression variation across the BXD hippocampus. A total of 71 strains were employed for hippocampal transcriptomic profiling, with two probes 1415904_at **(A)** and 1431056_a_at **(B)**, representing the *Lpl* expression level. The values are log2 transformed, and mean ± SE was used.

We examined the hippocampus transcriptome across 67 BXD lines plus the two parentals, B6 and D2, and the two F1 hybrids. Two probes target the *Lpl* gene body, with one probe (1415904_at) at the distal 3′ UTR region and one (1431056_a_at) targeting exons 7, 8, and 9. The average mRNA level of 1415904_at is 11.815 ± 0.612 SD, with the B6 and BXD99 mice having the lowest and highest expression of 10.768 and 12.219 (Figure 3A), respectively. For probe 1431056_a_at, the average mRNA level is 8.798 ± 0.453 SD, with the B6 and BXD99 mice having the lowest and highest expression of 8.079 and 9.994 (Figure 3B), respectively. Those two probes showed consistent expression patterns across the BXD mice, with a Pearson correlation *r* = 0.943 and *p*-value < 0.0001.

**FIGURE 4:**
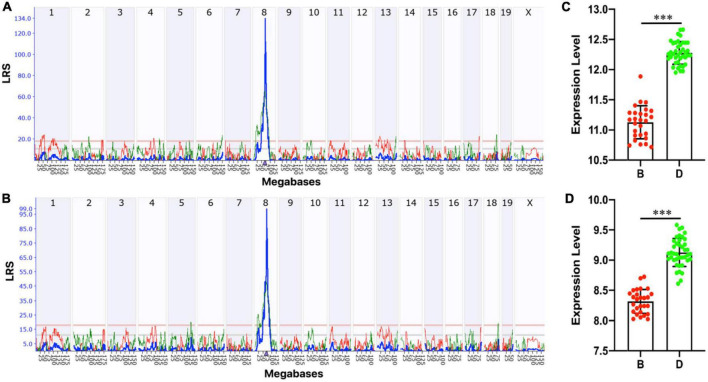
Expression quantitative trait locus (eQTL) mapping for *Lpl*. Manhattan plots showing the genome-wide regulating locus for probe 1415904_at **(A)** and 1431056_a_at **(B)** in the BXD hippocampus. The *x*-axis denotes a position on the mouse genome in megabases (Mb) and the *y*-axis indicates the LRS score. This analysis was done on GeneNetwork (https://www.genenetwork.org/) with the “interval mapping” method. The genome-wide suggestive and significant thresholds were determined with 1,000 permutation tests. Bar plots showing the mRNA level of probe 1415904_at **(C)** and 1431056_a_at **(D)** between **(B,D)** alleles at 69.676 Mb on Chr 8 (rs48549917). The values are log2 transformed and ***p < 0.0001.

Lpl is located on Chromosome (Chr) 8 at 68.9 Mb. To explore whether genomic loci regulate the *Lpl* expression variation, we performed a genome-wide eQTL mapping with the “interval mapping” method. Under the suggestive and significant threshold of 11.0 and 17.9 determined by 1,000 permutation tests, one significant eQTL for probe 1415904_at was mapped to Chr 8 at 69.676 Mb (rs48549917) with the peak LRS of 134.15 (Figure 4A). This locus is located at 0.1 Mb of Lpl, suggesting that *Lpl* is *cis*-regulated in the BXD hippocampus. The other probe, 1431056_a_at, was also mapped to the same locus (LRS = 99.056, Figure 4B). Moreover, we also found the same *cis*-QTL in several other BXD tissues, including the amygdala, brain, eye, midbrain, nucleus accumbens, pituitary, prefrontal cortex, spleen, and ventral tegmental area. Statistical analysis between the two cohorts grouped by the genotype at the QTL peak position (rs48549917) demonstrated that BXD mice with the D2 allele showed significantly higher *Lpl* expression than those mice carrying the B6 allele (*p* < 0.0001, Figures 4C, D).

### Identification of genetic variations of Lpl

*Lpl* is *cis*-regulated, which means that sequence variants within or nearby *Lpl* likely affect its expression. Therefore, we explored the genetic variations on the Mouse Genome Project database, in which the whole genome was sequenced over 30 classical inbred strains, including B6 and D2. As shown in Table 2, we identified 27 SNPs at the 3′ UTR region, one SNP at the 5′ UTR region, and three SNPs at the splice region. Besides, eight SNPs were located at the Lpl coding region, with two (rs48623874, rs33121577) defined as missense variants and the other six as synonymous variants.

**TABLE 2:** Lists of Lpl genetic variants between B6 and D2.

Chr	Position	dbSNP	Ref	DBA/2J	Type	TF
8	68904539	rs46345856	T	C	3_prime_utr	NHLH2
8	68904738	rs48259087	T	C	3_prime_utr	NA
8	68904747	rs8253499	G	T	3_prime_utr	Zic2, ZNF317, Zic3
8	68904797	rs50930818	C	T	3_prime_utr	NA
8	68904931	rs49173156	A	G	3_prime_utr	NA
8	68904952	rs47929877	T	C	3_prime_utr	DUX
8	68904954	rs50307489	A	G	3_prime_utr	NA
8	68905178	rs244771561	A	G	3_prime_utr	ATF7, CDX1, CDX2, CDX4, HOXD10, HOXC12
8	68905238	rs254737771	G	A	3_prime_utr	NA
8	68905258	rs50958623	A	G	3_prime_utr	MSANTD3
8	68905297	rs49436803	A	C	3_prime_utr	NA
8	68905347	rs232011516	G	A	3_prime_utr	ZNF282
8	68905376	rs52439029	T	C	3_prime_utr	NA
8	68905792	rs13470201	C	A	3_prime_utr	NR5A1, NR5A2
8	68905874	rs108904688	T	C	3_prime_utr	NA
8	68906389	rs8236728	G	A	3_prime_utr	ZNF331, ZNF682, ZNF449
8	68906598	rs8253498	C	T	3_prime_utr	NA
8	68907303	−	A	G	3_prime_utr	NA
8	68907333	rs33449771	G	A	3_prime_utr	MAFF,NRL
8	68907336	rs33176327	G	A	3_prime_utr	NRL, NR2F1, CTCF
8	68907355	rs50695992	C	T	3_prime_utr	NA
8	68907378	rs48718417	A	T	3_prime_utr	NA
8	68904980	rs262678694	G	GT	3_prime_utr	NA
8	68905231	rs229666878	G	GGATAGATGTTGAAAAT	3_prime_utr	NA
8	68905549	-	GA	G	3_prime_utr	NA
8	68905725	rs222423431	ACTT	A	3_prime_utr	SPIB, SPI1
8	68906070	rs229841610	AT	A	3_prime_utr	MSGN1, YY1
8	68880605	rs32769281	T	C	5_prime_utr	NA
8	68899458	rs48623874	G	A	Missense	NA
8	68901238	rs33121577	A	G	Missense	NA
8	68887444	rs33396764	G	A	Splice_region	NA
8	68899545	rs51454640	T	C	Splice_region	NA
8	68891362	rs257133866	CTTAAAATCG	C	Splice_region	NA
8	68895786	rs33408109	A	G	Synonymous	NA
8	68895801	rs32832821	A	G	Synonymous	NA
8	68896627	rs33594984	C	T	Synonymous	NA
8	68896684	rs33075533	G	A	Synonymous	NA
8	68899487	rs48000521	G	A	Synonymous	NA
8	68901262	rs49765636	T	C	Synonymous	NA

We predicted the two missense variants’ functional effects on protein function with SIFT in Variant Effect Predictor (McLaren et al., 2016) and found that rs33121577 is a deleterious amino acid substitution (c.1492 A > G, p.I410M, SIFT = 0.03). We further speculate that these UTR mutations may be located at transcription factor (TF) binding motifs and affect Lpl gene expression by altering the TF binding capacity. To confirm this, we looked up the variants in the JASPAR, an open-access database storing manually curated TF binding profiles (Castro-Mondragon et al., 2022), and found that several variants hit the TF binding motifs (Table 2), including rs46345856 (NHLH2), rs8253499 (ZIC2, ZNF317, ZIC3), rs47929877 (DUX), rs244771561 (ATF7, CDX1, CDX2, CDX4, HOXD10, HOXC12), rs50958623 (MSANTD3), rs232011516 (ZNF282), rs13470201 (NR5A1, NR5A2), rs8236728 (ZNF331, ZNF682, ZNF449), rs33449771 (MAFF, NRL), rs33176327 (NRL, NR2F1, CTCF), rs222423431 (SPIB, SPI1), and rs229841610 (MSGN1, YY1).

### Genetic correlations between *Lpl* and hippocampal transcriptome

**FIGURE 5:**
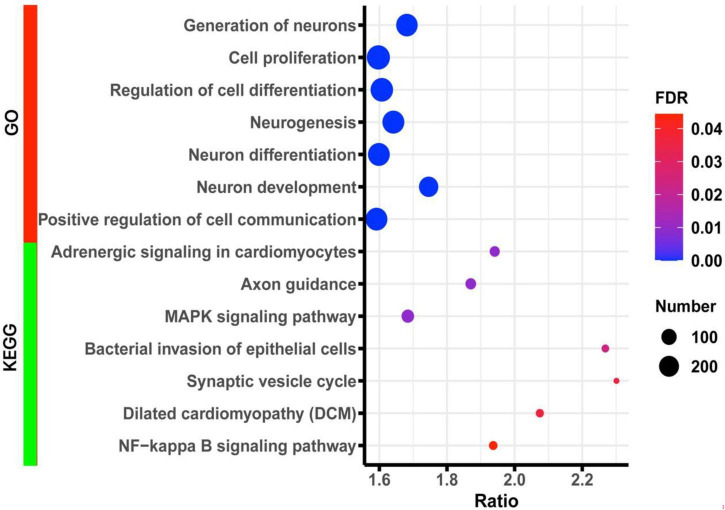
Bubble charts of the enrichment results of GO and KEGG terms for the *Lpl* correlated genes. The *x*-axis represents an enriched ratio, and the *y*-axis represents enriched terms. The size of the dots represents the number of genes, and the color indicates the FDR value. An enriched ratio is defined as the number of observed divided by the number of expected genes from the annotation category in the gene list.

To further understand the underlying biological processes and pathways involved in *Lpl*, we performed a Pearson correlation analysis between *Lpl* (1415904_at) and the other hippocampal genes. This resulted in 3,758 probes (corresponding to 3,364 transcripts) that are significantly (*p* < 0.05) correlated with the *Lpl* expression. The gene set enrichment analysis showed that those genes were significantly (FDR < 0.05) enriched in the GO biological processes of cell proliferation (291 genes), generation of neurons (243 genes), neurogenesis (254 genes), neuron differentiation (220 genes), and neuron development (185 genes) (Figure 5). For KEGG pathways, those genes were significantly (FDR < 0.05) involved in the MAPK signaling pathway (59 genes), axon guidance (39 genes), synaptic vesicle cycle (17 genes), and NF-kappa B signaling pathway (24 genes) (Figure 5).

Previous results have demonstrated that the synaptic vesicle cycle could be mediated by memory impairment and presynaptic dysfunction in the Lpl-deficient mice. Therefore, we further explored those 17 *Lpl*-correlated genes involved in the synaptic vesicle cycle. Among these, *Atp6v1b2* (*r* = 0.550 and *p* = 7.69 E-07) and *Rab3a* (*r* = −0.374 and *p* = 0.002) show the most positive and negative correlation with *Lpl*, respectively.

**FIGURE 6:**
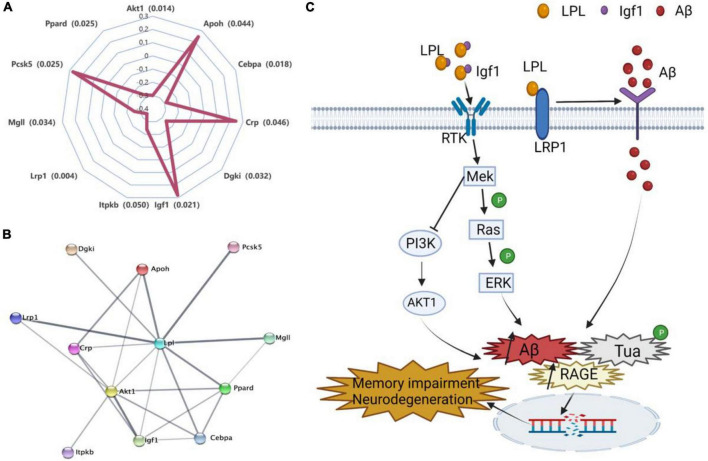
Molecular mechanisms of Lpl involved in the regulation of cognitive dysfunction. **(A)** Correlations between the expression of *Lpl* and its directly interacted genes. The Pearson correlation coefficient was used to determine the relationship. **(B)** The Lpl PPI interaction network. The network was created and evaluated with the 11 Lpl directly interacted genes using string (https://www.string-db.org/). **(C)** Lpl interacts with Igf1 to activate the MAPK signaling pathway while blocking the PI3K/AKT1 signaling pathway, leading to amyloidβ (Aβ) toxicity, increased RAGE expression, tau hyperphosphorylation, induction of apoptosis, and autophagy. Lrp1 regulates Aβ binding and uptake in neurons and interacts with Lpl to activate the MAPK signaling pathway, inducing neuronal damage that leads to cognitive dysfunction.

To narrow down the Lpl potentially directly interacted genes, we searched those genes in the string website, a database of known and predicted protein–protein interactions. We identified 11 genes (Akt1, Apoh, Cebpa, Crp, Dgki, Itpkb, Lrp1, Mgll, Pcsk5, and Ppard) directly connected to Lpl (Figures 6A, B), especially for Igf1, Akt1, and Lrp1. In neurons, Lpl binds to Igf1 and activates the MAPK signaling pathway along with the PI3K/AKT1 signaling pathway, leading to amyloid β (Aβ) toxicity, increased RAGE expression, tau hyperphosphorylation, induction of apoptosis, and autophagy (Figure 6C). LRP1 regulates Aβ binding and uptake in neurons and interacts with Lpl to regulate energy homeostasis and cognitive function (Figure 6C).

## Discussion

In this study, we demonstrate, among the 280 genes related to lipid metabolism, that the Lpl plays a pivotal role in regulating cognitive function, as evidenced by the significant correlations with all four cognition-related traits. Specifically, we found a negative correlation between *Lpl* expression and latency to reach a hidden platform, indicating impaired cognitive function with a lower level of *Lpl* expression. Supporting our results, cognitive decline was observed in an Lpl deficiency mouse model, including increased latency to an escape platform and increased mistake frequency in a water maze test, and decreased latency to a platform in the step-down inhibitory avoidance test (Xian et al., 2009). In addition, our PheWAS also indicated the association of Lpl genetic polymorphisms with ventral hippocampus volume residuals and several anxiety-related behavioral traits. These findings align with the ventral hippocampus function that is involved in the control of emotional and anxious behaviors (Gulyaeva, 2015). Thus, our gene–phenotype correlation and genotype–phenotype association analysis demonstrated that Lpl is associated with cognitive dysfunction as well as emotional and anxious behaviors through modulation of hippocampus functions.

As a lipoprotein metabolism risk gene, Lpl plays a critical role in breaking down fat in the form of triglycerides, which are carried from various organs to the blood *via* lipoprotein molecules. LPL mRNA is found predominantly in the hippocampus and is 2.5-fold higher than in other brain regions (Wang and Eckel, 2012). A recent study has reported that AD is highly associated with LPL featuring on CNS microglia linked with phagocytosis and protection of AD (Hemonnot et al., 2019). In addition, Lpl-deficient mice displayed some degree of memory impairment and presynaptic dysfunction (Liu et al., 2014). In humans, LPL is strongly *cis*-regulated in whole blood, lung, spleen, thyroid, adipose, testis, and brain (GTEx Consortium, 2020). Moreover, dozens of pathogenic variants have been identified in its gene body (Landrum et al., 2020). Genetic mutations in LPL have been associated with AD risk (Bruce et al., 2020), especially clustered with other cholesterol-related gene mutations (Papassotiropoulos et al., 2005). In the current study, we systemically analyzed the *Lpl* expression and genetic regulation in the hippocampus in the BXD family mice. Consistent with the previous findings, *Lpl* is highly expressed in the hippocampus and shown 2.7–3.7-fold change among the strains (Figure 3). By performing eQTL analysis, we confirmed that this variation is regulated by the local genetic variants (Table 2), in which BXD mice with the D2 allele showed significantly higher *Lpl* expression than those mice carrying the B6 allele (Figure 4). In addition, we also found the same *cis*-eQTL in several other tissues, demonstrating the robustness of this *cis*-regulation in the BXD family.

A gene set enrichment analysis was performed to evaluate *Lpl* pathways in the hippocampus. We observed that *Lpl* covariates are mainly involved in neuron development, neurogenesis, and neuron differentiation, as well as in several signaling pathways, including MAPK signaling pathway, axon guidance, synaptic vesicle cycle, and NF-kappa B signaling pathway (Figure 5). In neurons, synaptic vesicles are a class of small, electron-lucent vesicles that store various neurotransmitters released at the synapse, and are involved in the impairment of learning and memory function (Kennedy, 2016). A previous study showed that disruption of the synaptic vesicle cycle leads to presynaptic dysfunction and plasticity damage in LPL-deficient neurons (Liu et al., 2014). In the current study, we identified 17 *Lpl* covariates related to the synaptic vesicle cycle, with *Rab3a* showing the most negative correlation with *Lpl*, suggesting that Rab3a may mediate the negative regulation of synaptic vesicle cycles by Lpl. Ras-associated binding protein 3A (Rab3a) is a neuronal guanosine triphosphate binding protein that binds synaptic vesicles and regulates synaptic transmission. In relation to our findings, increased hippocampal *Lpl* expression was observed in *Rab3a*^–/–^ mice (Yang et al., 2007). As an important transmitter of extracellular information from the cell surface to the intracellular space, MAPK signaling has been implicated in AD with various mechanisms, including amyloid beta (Aβ) toxicity, increasing RAGE expression, tau hyperphosphorylation, induction of apoptosis, and deregulated autophagy (Kheiri et al., 2018). In this study, we identified serine/threonine kinase 1 (Akt1) and insulin-like growth factor-1 (Igf1), part of the enriched MAPK pathway, that directly interacted with Lpl through PPI network analysis. Studies have shown that impaired insulin signaling pathways regulate amyloid precursor protein processing (Adlerz et al., 2007) and Aβ clearance by blocking PI3K/AKT pathway, which may partially explain why diabetic patients are susceptible to AD (Sun et al., 2020).

The resulting PPI network also showed that several direct interactors of Lpl were involved in neuron-related functions, such as Crp and Lrp1 (Figure 6). CRP has been reported to have an essential clinical significance in cardiovascular disease and AD (Luan and Yao, 2018). In addition, CRP has also been linked with the activation of the MAPK signaling pathway in AD (Cargnello and Roux, 2011). In neurons, LRP1 can regulate cellular Aβ binding and uptake (Liu et al., 2017). Furthermore, LRP1 deficiency in forebrain neurons leads to disturbances in brain lipid metabolism, progressive and age-dependent synaptic loss, memory loss, and neurodegeneration (Liu et al., 2010). These phenotypes are similar to those in LPL-deficient mice (Xian et al., 2009). In the hypothalamus, LRP1^–/–^ mice exhibit obesity associated with hyperlipidemia, glucose intolerance, and insulin resistance (Liu et al., 2011), with similar phenotypes observed in neuronal LPL-deficient mice (Wang et al., 2011). These results suggest that LPL and LRP1 interact in some way in brain to regulate energy homeostasis and cognitive function.

In summary, by taking advantage of the BXD family mice for genomic, phenomic, and hippocampal transcriptomic data, our results indicate that Lpl is associated with cognitive dysfunction-related phenotypes. The co-expression and PPI network analysis revealed that Lpl participates in cognition function through the MAPK signaling pathway and synaptic vesicle cycle, and by directly interacting with the neuron function-related gene *Rab3a*, Akt1, Igf1, Crp, and Lrp1 (Figure 6). Our findings demonstrate the importance of the Lpl, among the cholesterol-related genes, in regulating cognitive dysfunction, which may serve as a novel therapeutic target for treating cognitive dysfunction.

## Data availability statement

The datasets presented in this study can be found in online repositories. The names of the repository/repositories and accession number(s) can be found below: https://www.ncbi.nlm.nih.gov/geo/, GSE84767.

## Ethics statement

All procedures involving mouse tissue were approved by the Institutional Animal Care and Use Committee at the University of Tennessee Health Science Center.

## Author contributions

FX and GT conceived the study. WH and JL conducted and performed the data analysis. WH, JL, JM, and FX wrote the manuscript. YH, QX, TD, and MW prepared the figures and tables. JB, LL, and GT edited the manuscript. All authors read and approved the final version of the manuscript for publication.

## Footnotes

1
https://www.ncbi.nlm.nih.gov/geo/

2
http://gn1.genenetwork.org/webqtl/main.py?FormID=sharinginfo and GN_AccessionId=600 and InfoPageName=BXDGeno

3
https://www.sanger.ac.uk/data/mouse-genomes-project/

4
http://www.webgestalt.org/

5
https://systems-genetics.org/

6
http://amigo.geneontology.org/amigo
